# Role of G Protein-Coupled Receptors in the Regulation of Structural Plasticity and Cognitive Function

**DOI:** 10.3390/molecules22071239

**Published:** 2017-07-24

**Authors:** Crystal C. Y. Leung, Yung H. Wong

**Affiliations:** 1 Division of Life Science, Biotechnology Research Institute, Hong Kong University of Science and Technology, Clear Water Bay, Kowloon, Hong Kong, China; cycleung@connect.ust.hk; 2 State Key Laboratory of Molecular Neuroscience, Molecular Neuroscience Center, Hong Kong University of Science and Technology, Clear Water Bay, Kowloon, Hong Kong, China; 3 Guangdong Provincial Key Laboratory of Brain Science, Disease and Drug Development, HKUST Shenzhen Research Institute, Shenzhen 518057, Guangdong, China

**Keywords:** G protein, GPCR, hippocampus, structural plasticity, synapse

## Abstract

Cognition and other higher brain functions are known to be intricately associated with the capacity of neural circuits to undergo structural reorganization. Structural remodelling of neural circuits, or structural plasticity, in the hippocampus plays a major role in learning and memory. Dynamic modifications of neuronal connectivity in the form of dendritic spine morphology alteration, as well as synapse formation and elimination, often result in the strengthening or weakening of specific neural circuits that determine synaptic plasticity. Changes in dendritic complexity and synapse number are mediated by cellular processes that are regulated by extracellular signals such as neurotransmitters and neurotrophic factors. As many neurotransmitters act on G protein-coupled receptors (GPCRs), it has become increasingly apparent that GPCRs can regulate structural plasticity through a myriad of G protein-dependent pathways and non-canonical signals. A thorough understanding of how GPCRs exert their regulatory influence on dendritic spine morphogenesis may provide new insights for treating cognitive impairment and decline in various age-related diseases. In this article, we review the evidence of GPCR-mediated regulation of structural plasticity, with a special emphasis on the involvement of common as well as distinct signalling pathways that are regulated by major neurotransmitters.

## 1. Introduction

**Figure 1:**
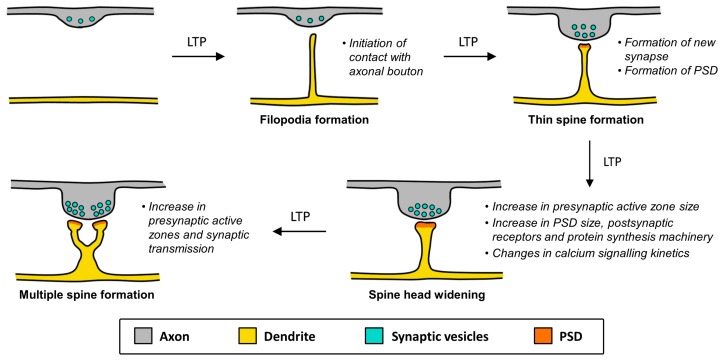
Spine morphogenic effects of long-term potentiation (LTP). Dendritic spines sprout and alter their morphology in an activity-dependent manner. LTP leads to the protrusion of filopodia and immature spines from the dendritic shaft and eventually spine maturation. Multiple spines can also synapse onto the same axonal bouton. These morphological changes are accompanied by alterations in cell physiology that impact synaptic transmission, which include the formation of the postsynaptic density (PSD), changes in the quantity of synaptic vesicles and postsynaptic neurotransmitter receptors, and changes in calcium compartmentalisation.

The average human brain has around 100 billion neurons that are connected with each other via specialised structures known as synapses. As each neuron may form synapses with up to 10,000 other neurons, it is almost impossible to fathom the complexity associated with higher brain functions such as cognition. Synapses are in fact critical sites for memory storage. There is ample evidence to demonstrate that synaptic plasticity (the strengthening or weakening of existing synapses) and structural plasticity (the remodelling or formation of spines) underlie learning and memory [1,2]. In particular, the latter allows for the activity-dependent formation of new synapses, and provides the basis for neural circuits to be rewired in response to experiences or changes in the environment [1]. Structural and synaptic plasticity are actually closely intertwined. Sprouting of spines and morphological changes often occur after long-term potentiation (LTP) [3,4], and such changes in spine number or spine geometry could impact synaptic density or the magnitude of postsynaptic chemical responses (Figure 1).

Structural plasticity is mostly studied in the dendritic spines of the postsynaptic neurons, which are the principal recipients of excitatory input, as they contact axonal boutons to form synapses. These microscopic neuronal structures are intimately associated with memory processes, with the spine morphology and density being altered on an experience-dependent basis [1]. For example, exposure of adult male rats to a spatially complex environment significantly increased the spine density on CA1 pyramidal cells in the hippocampus, which was indicative of new excitatory synapse formation [5]. Changes in spine morphology can also be observed when animals acquire memory through different conditioning paradigms [6,7] or through training for the Morris water maze task [8]. Such structural changes in the actin-rich dendrite spines have been proposed to be one of the key mechanisms underlying long-term memory formation [9,10].

Spine morphogenesis requires a reorganisation of the actin cytoskeleton in dendritic spines through the action of actin-binding proteins (ABPs). For instance, the Arp2/3 complex promotes nucleation of actin filaments to create a branched actin network characteristically found in spine heads [11], while cofilin stimulates actin depolymerisation and severing for spine remodelling, and profilin promotes the opposite [12]. Many of the upstream signals modulating cytoskeletal dynamics converge upon the regulation of small GTPases to exert their effects. The Rho family of small GTPases [13] is often involved in the process, with Ras homologue family member A (RhoA) negatively regulating actin polymerisation, and the Ras-related C3 botulinum toxin substrate 1 (Rac1) and cell division control protein 42 homolog (Cdc42) driving filamentous actin (F-actin) formation.

Cell surface receptors on postsynaptic neurons are stimulated by different extracellular signals to drive spine morphogenesis. Much of the focus has been placed on the glutamatergic ionotropic receptors, receptor tyrosine kinases and cell adhesion molecules [1]. However, many other neurotransmitters that act on G protein-coupled receptors (GPCRs) are equally effective in modulating neuronal excitability and structure. Out of the 800 or more GPCRs, 353 are non-odorant receptors, of which over 76% can be found in the hippocampus, according to an mRNA expression profiling study conducted in adult mouse tissue [14].

**Figure 2:**
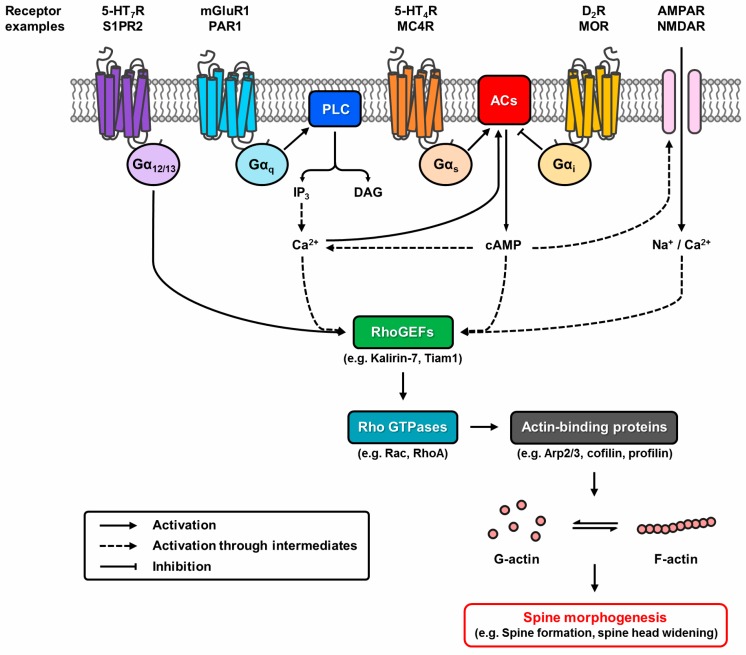
Putative pathways of G protein-coupled receptor (GPCR) modulation of structural plasticity. Dynamic reorganisation of the actin cytoskeleton controlled by actin-binding proteins underlies spine morphogenesis. GPCRs modulate this event by regulating Rho GTPase activity through Rho guanine nucleotide exchange factors (RhoGEFs). G_12/13_-coupled receptors directly interact with RhoGEFs, whereas G_q_- and G_s/i_-coupled receptors alter the activation state of RhoGEFs via the second messengers Ca^2+^ or cyclic adenosine monophosphate (cAMP). Crosstalk potential exists as G protein signalling pathways overlap. GPCRs can also modify the Ca^2+^ permeability of glutamatergic ion channels to modulate the process.

While more than 20 of the GPCRs abundantly expressed in the hippocampus have been demonstrated to mediate synaptic plasticity, less attention has been devoted to their potential role in structural plasticity. However, given the tight association between synaptic and structural plasticity, it is not uncommon for GPCRs to modulate both processes (Table 1). With more evidence linking GPCRs to structural plasticity emerging in recent years, it is timely to review the role of classical G protein signalling and other unconventional GPCR involvements in spine morphogenesis (Figure 2). Receptors expressed postsynaptically in the hippocampus and the cortical regions, which are brain areas associated with high plasticity, are of particular interest, given that actin dynamics are tightly regulated in a spatiotemporal manner via Rho GTPases. GPCRs that are more intricately linked to learning and memory, including the 5-hydroxytryptamine receptors (5-HTRs) and metabotropic glutamate receptors (mGluRs), will be discussed as well. As spine morphogenesis is studied more extensively in the neurodevelopmental context, this review will also draw upon such evidence, as these findings are potentially germane to structural plasticity.

## 2. Involvement of G Protein-Coupled Receptors in Spine Morphogenesis via Classical and Unconventional Pathways

### 2.1. The Cyclic Adenosine Monophosphate-Dependent Pathway—G_s_/G_i_ Signalling

G_s_ proteins stimulate adenylyl cyclase (AC) to produce intracellular cyclic adenosine monophosphate (cAMP), whereas G_i_ proteins inhibit the same process. The protein kinase A (PKA) activity elicited by cAMP and the subsequent activation of the cAMP response element binding protein (CREB) are well-established to be associated with the late phase of LTP and long-term memory storage [29]. It has been demonstrated that the phosphorylation, or activation, of CREB leads to a significant increase in spine density on the cultured hippocampal neuron [30]. This effect is mimicked by PKA-specific agonists such as Sp-Adenosine 3′,5′-cyclic monophosphorothioate (Sp-cAMP[S]), but is attenuated upon inhibition of PKA by H-89, indicating a potential role for G_s_ signalling in spine morphogenesis. Also, by phosphorylating the GluN1 subunit of the *N*-methyl-d-aspartate receptor (NMDAR) [31], PKA promotes Ca^2+^ permeability of the receptor [32], which allows for the activation of Ca^2+^-sensitive AC to amplify the initial extracellular signal. Indeed, more than 15 G_s_-coupled receptors have been shown to modulate memory processes, with ligands spanning a variety of hormones, including the corticotropin-releasing hormone [33], estrogen [34], prostaglandin E [35], and vasoactive intestinal peptide [36]; neurotransmitters such as adrenaline [37], γ-aminobutyric acid (GABA) [38], and glutamate [38]; and neuropeptides such as neuropeptide S [39].

In 1976, serotonin was shown to enhance synaptic transmission through increasing cAMP levels in the classical conditioning of *Aplysia* [40], making it one of the earliest neuromodulators found to mediate short-term memory and memory consolidation. The G_s_-coupled 5-HT receptors, 5-HT_4_R, 5-HT_6_R and 5-HT_7_R, are expressed in the hippocampus with evidence supporting their respective involvement in synaptic plasticity [41,42,43]. In particular, 5-HT_4_R and 5-HT_7_R are capable of increasing spine density, albeit via distinct mechanisms (Table 1). Activation of the G_s_-coupled 5-HT_4_R was shown to enhance the performance of adult male mice in a simultaneous olfactory discrimination task [16]. This effect was PKA-dependent and apparently coupled with more prominent learning-induced spine growth on CA1 pyramidal cells over the control. In contrast, 5-HT_7_R acts through G_12/13_ signalling and will be discussed below.

Other G_s_-coupled receptors also feature prominently in the regulation of memory encoding. D_1_-like dopamine receptors modulate the persistence of long-term fear [44] and spatial memories [45] in the hippocampus by integrating inputs from the motivation center, the ventral tegmental area (VTA). However, neither overexpression nor knockdown of the D_1_ receptor (D_1_R) had any effect on the spine density of hippocampal neurons in mice [20]. The adrenergic system that activates β-adrenergic receptors (β-ARs) is another key regulator of synaptic plasticity in the hippocampus. The β-ARs potentiate LTP in the dentate gyrus and the CA3 regions, and stimulate the mitogen-activated protein kinases/extracellular signal-regulated kinases (ERK) signalling cascade through the cAMP-dependent pathway to promote protein synthesis, a critical step for late-LTP and long-term memory formation [46]. However, there is little support at present for a role of β-ARs in structural plasticity. The adenosine receptors are linked to cognition as well, notably being associated with the memory-enhancing effects of caffeine, which acts as an antagonist of the receptor family [47]. The adenosine A_2A_ receptor (A_2A_R) facilitates LTP in the hippocampus through the classical G_s_ signalling pathway [48], while the G_i_-coupled A_1_R exerts a tonic inhibitory effect on the process [49]. Despite this, the relationship between adenosine receptors and spine morphogenesis remains elusive to date.

While the α-melanocyte-stimulating hormone (α-MSH) is best known in the brain for its regulation of energy metabolism, its cognate receptor, melanocortin-4 receptor (MC4R), was recently shown to also facilitate structural plasticity. Agonist activation of MC4R produced an increase in the number of mature spines, whereas knockdown of the receptor caused a fall in spine density and a decline in the proportion of spines with a mushroom head [22]. The loss of mature spines could only be rescued by re-expression of wild-type MC4R, but not the MC4R point mutant that is unable to interact with Gα_s_. The involvement of G_s_ signalling is thus implicated in MC4R’s ability to alter spine morphology.

The intracellular cAMP level is an outcome of the opposing G_s_ and G_i_ signals at play. It is therefore important not to neglect the significance of G_i_-coupled receptors in regulating spine morphogenesis. Studies pertaining to this group of GPCRs are nevertheless relatively limited. It has been reported that, in adolescent but not adult mice, agonists of the dopamine D_2_ receptor (D_2_R) reduce spine density on hippocampal CA1 neurons in a manner that depends negatively on cAMP and requires the internalisation of an NMDAR subunit, GluN2B [20]. These pharmacological observations were confirmed through in vivo experiments. Overexpression of D_2_R led to a decrease in spine density, whereas receptor knockdown produced more spines. Morphologically, D_2_R activation appeared to hinder spine maturation, as the density of filopodia increased and the spines saw an elongation of neck length [20].

The opioid receptors have also drawn interest from investigators, owing to the cognitive impairments associated with sustained opioid usage [50,51], and because chronic exposure to morphine reduces the spine density of CA1 hippocampal neurons in mice [52]. A separate study using cultured rat hippocampal neurons demonstrated that morphine’s interference with spine stabilisation could be countered by treatment with CTOP (D-Phe-Cys-Tyr-D-Trp-Orn-Thr-Pen-ThrNH2), a µ-opioid receptor (MOR) antagonist, or knockout of MOR [25]. Even though G_i_ signalling was not explicitly shown to mediate this effect, the authors concluded that the spine morphogenic effects were not due to altered neuronal activity, as morphine retained its impact on spine turnover even when co-treated with tetrodotoxin, a sodium channel blocker. It is of interest to note that AC superactivation occurs as a result of sustained G_i_ signalling downstream of persistent MOR activation [53,54]. Such homeostatic adjustment by the cell signalling machinery leads to cAMP accumulation upon opioid withdrawal, and could potentially drive spine morphology changes in the opposite direction.

Like opioids, cannabinoids act on G_i_-coupled receptors, and are known for their analgesic function and dependence issues. Interestingly, the cannabinoid type 1 receptor (CB_1_R) appears to impact spine stability as well. Activation of CB_1_R in mature mice cortical neurons led to a significant decrease in spine density that was attributable specifically to a drop in the number of mushroom spines [19]. The authors showed that CB_1_R agonists encouraged F-actin disassembly in wild-type mice, but actin dynamics remained unaffected in CB_1_R knockout mice. Co-immunoprecipitation results indicated that CB_1_R interacts with components of the Wiskott–Aldrich syndrome protein family verprolin-homologous protein 1 (WAVE1) complex and negatively regulates WAVE1 disinhibition in a manner that is dependent on Rac1 and G_i_ signalling. Such results are in line with the observation that WAVE1 promotes actin nucleation downstream via the Arp2/3 complex [55].

### 2.2. G_12/13_ Signalling

The G_12_ subfamily, comprising the Gα_12_ and Gα_13_ subunits, regulates another key pathway for GPCR-mediated structural plasticity. These Gα subunits directly interact with RhoGEFs to activate the monomeric Rho GTPases [56] and eventually modulate actin dynamics via ABPs, making them obvious candidates for a role in spine morphogenesis. In cultured Gα_12_-knockout hippocampal neurons, a significant decrease in dendritic spine protrusions compared to wild-type was observed [17]. In the same study, 5-HT_7_R, previously shown to couple to Gα_12_ [57], was found to promote the formation of short protrusions and filopodia in wild-type neurons and hippocampal slices (Table 1). This effect was abolished upon knockout of Gα_12_, in 5-HT_7_R siRNA treatment, or under both conditions in dissociated neuronal cultures [17]. This was consistent with earlier experiments indicating that 5-HT_7_Rs are able to induce filopodia formation via Cdc42 (which lies downstream of G_12_) in NIH3T3 cells [57]. RhoA, whose inhibition is required for neurite outgrowth [58], can also be stimulated by 5-HT_7_Rs [57]. This Ca^2+^-sensitive activity, however, may be suppressed in neurons, as receptor activation also causes a transient decline in postsynaptic Ca^2+^ levels. In fact, 5-HT_4_ receptors are shown to couple to Gα_13_ [59], which interacts with the p115 RhoGEF to activate RhoA [60]. Interestingly, contrary to the stimulatory outcome on dendritic spine growth in a previous study [16], the authors observed rounding of cells and neurite retraction following agonist stimulation in NIE-115 cells [59].

Another G_13_-coupled receptor, the sphingosine-1-phosphate receptor 2 (S1PR2), was found to maintain dendritic spine stability when activated by the membrane protein Nogo-A. Pharmacological blockade of S1PR2 led to spine formation within 3 h in mice CA3 pyramidal neurons, whereas agonist treatment elicited a decrease in the average spine length and spine head width that could be neutralised by inhibiting the Rho-associated, coiled-coil-containing protein kinase (ROCK) that lies downstream of RhoA activation [28]. A separate study presented evidence of Nogo-A/S1PR2 signalling through Gα_13_, which activates the leukemia-associated RhoGEF (LARG) to stimulate RhoA [27], potentially allowing Nogo-A to exert its effects by deactivating cofilin [61].

An adhesion GPCR, brain-specific angiogenesis inhibitor 1 (BAI1), was also demonstrated to activate RhoA via a G_12/13_-dependent mechanism [62], and a separate study showed that receptor knockdown in rat hippocampal neurons resulted in a decrease in spine density and more immature spines [18]. Independently, BAI1-knockout mice were found to harbour deficits in spatial learning and memory [63]. As constitutive G_12_ activity was observed with 5-HT_7_ receptor overexpression [57], it is possible that BAI1’s targeted expression in dendritic spines [18] could promote spine morphogenesis and mediate cognitive functions through the same pathway.

While the two G_12_ family members are often studied together, these Gα subunits share a mere 65% of their amino acid sequence identity in humans, and possess differential signalling and phenotypic characteristics [64]. For instance, Gα_12_-deficient mice are viable, but those lacking Gα_13_ typically do not survive past embryonic day 10 [65]. Gα_13_ can activate Rho via p115 RhoGEF, but the same is not observed for Gα_12_ [60]. Activation of the G_12_-coupled 5-HT_7_R lengthens neurites through Cdc42 [57]. On the other hand, 5-HT_4_R acts via Gα_13_ and RhoA to cause neurites to retract and decrease in number [59]. Given the selectivity of Rho GTPase activation and the antagonistic phenotypes produced, further studies delineating the respective signalling properties of Gα_12_ and Gα_13_ may prove to be interesting.

### 2.3. G_q_ Signalling

Ca^2+^ is a key second messenger in active dendritic spines [66]. The accumulation of Ca^2+^ drives a cascade of signalling activity in response to synaptic activation. In LTP, Ca^2+^ turns on calcium/calmodulin-dependent protein kinase II (CaMKII), which then phosphorylates RhoGEFs to exert control on actin dynamics and, hence, spine morphogenesis [67]. Low frequency stimulation of neurons, however, turns on the phosphatase calcineurin to promote actin depolymerisation by dephosphorylating cofilin [68]. Moreover, it is speculated that different sources of Ca^2+^ induce distinct changes in spine morphology. The release of Ca^2+^ from intracellular stores is associated with spine elongation or formation [69], whereas an influx of Ca^2+^ (e.g., via glutamate-gated channels) leads to the collapse of spines [70]. G_q_ signalling involves the activation of phospholipase C-β, followed by that of protein kinase C (PKC) and the release of intracellular Ca^2+^ stores. At least nine G_q_-coupled receptors expressed in the hippocampus are found to regulate memory processes; they include those from the 5-HT [71], mGluR [72] and protease-activated receptor (PAR) families [73]. These GPCRs are also well-positioned to mediate spine modifications with their ability to cause transient increases in intracellular Ca^2+^ levels (Table 1).

In rat hippocampal slices and dissociated neurons, global activation of G_q_-coupled group I mGluRs via agonist treatment induced an elongation of thin and filopodia-like dendritic spines with a limited change in spine density [23]. Calcium chelation or protein synthesis blockade, but not NMDAR or α-amino-3-hydroxy-5-methyl-4-isoxazolepropionic acid receptor (AMPAR) inactivation, diminished such lengthening. Consistent with the hypothesis that differential Ca^2+^ stores produce different effects on spine morphology, group I mGluRs appear to also mediate activity-dependent spine shrinkage when activated alongside NMDARs. In rat CA1 pyramdial neurons that were subjected to low-frequency glutamate uncaging, pharmacological blockade of group I mGluRs attenuated the decrease in spine length. It was further shown that this effect requires the activation of inositol trisphosphate receptors (IP_3_Rs) that gate the release of Ca^2+^ from the endoplasmic reticulum (ER), but not PKC [74]. In particular, mGluR-dependent shrinkage was found to be specific to large spines. The authors suggested that this could possibly be attributed to a lack of ER, and that the Ca^2+^ influx induced by NMDAR activation alone suffices due to the relatively higher NMDAR concentration in smaller spines.

It has also been shown that application of 2,5-dimethoxy-4-iodoamphetamine (DOI; a 5-HT_2_R agonist) causes a transient increase in spine size in rat cortical pyramidal neurons [15]. This observation is dependent on the presence of RhoGEF kalirin-7 and its downstream activation of the p21-activated kinase. The G_q_-coupled 5-HT_2A_R is thus implicated in spine remodeling, as it is colocalised with kalirin-7 in a subset of spines. Despite its stronger influence over mood and appetite rather than cognition [75], the closely related 5-HT_2C_R could also be responsible because it is a receptor for DOI and its subcellular distribution coincides in the postsynaptic region with postsynaptic density (PSD)-95 [76]. Since kalirin-7 requires phosphorylation by CaMKII in order to be activated [77], it is possible that G_q_ signalling driven by 5-HT_2A_R and 5-HT_2C_R underlies the increase in spine size.

In mice overexpressing the astrocyte-derived matrix metalloproteinase-1, PAR1 activation can also induce Ca^2+^ flux and produce an increase in spine density in the CA1 pyramidal neurons [26]. Genetic deletion of PAR1 blocks the observed rise in the intracellular Ca^2+^ concentration. This adds to the mounting biochemical and behavioural evidence that the astrocytic PAR1 mediates memory and neuronal plasticity in the hippocampus [73,78,79].

In contrast to the study on group I mGluRs [74], it has been demonstrated previously that PKC does have a role to play in spine morphogenesis [80]. ERK phosphorylation resultant from PKC activation leads to de novo formation of spines in cultured mouse hippocampal neurons. This effect is additive to the CREB and ERK activation mediated by NMDAR.

### 2.4. Gα-Independent Mechanisms: Gβγ, β-Arrestin, and Others

Recent studies have revealed that GPCRs regulate spine morphogenesis via mechanisms that are Gα-independent. The Gβγ complex is another central participant of GPCRs’ signalling activities. The frizzled-9 (Fzd9) receptor, which is selectively expressed in the hippocampus [81] and is related to visuospatial learning and memory [82], stimulates spinogenesis in cultured hippocampal neurons through the Gβγ complex [21] (Table 1). This activity is mediated by the Wnt-5a ligand through the non-canonical Ca^2+^ pathway, which activates CaMKIIα. Knockdown of Fzd9 and treatment with pertussis toxin or β-AR kinase carboxyl-terminus (βARKct), a Gβγ scavenger, all attenuated the increase in spine density.

On top of receptor desensitisation, the scaffolding protein β-arrestin is found to also promote G protein-independent signaling. GPCR activation can trigger the formation of a signalling complex, wherein the protein kinase B (Akt)/glycogen synthase kinase 3 β (GSK-3β) pathway is activated [83]. Evidence suggests that GSK-3β activity increases the number of thin spines in the dentate gyrus [84]. In a previous study involving PAR1 [26], the authors postulated that β-arrestin could also underlie the observed spinogenesis, as the transgenic animals also displayed a decrease in the level of phosphorylated GSK-3β, consistent with reports that PAR1 activates β-arrestin signalling. This could potentially initiate spine remodelling, as β-arrestins are capable of forming signalling scaffolds to dephosphorylate, and thus activate, cofilin under stimulation of PAR2 [85]. Recruitment of β-arrestin following agonist stimulation is facilitated by GPCR kinases (GRKs) that phosphorylate the intracellular residues of the receptor to create a binding surface. In fact, GRKs are also found to modulate neurotransmission [86,87] and actin reorganisation [88]. GRK2, which can be found in the hippocampal regions, phosphorylates ezrin to enable membrane ruffling [88], a process that mirrors dendritic spine formation in that actin-rich protrusions are formed through Rac-mediated actin polymerisation. Interestingly, GRK2 is also shown to be responsible for the desensitisation of GPCRs that regulate structural plasticity, for example, the group I mGluRs [89,90] and S1PR2 [91]. Such evidence presents further potential for GRK involvement in spine morphogenesis, in addition to its integral role in keeping GPCR activity in check.

GPCRs can also exert their function by localising small GTPase activity to synaptic sites. As mentioned previously, the orphan adhesion GPCR, BAI1, regulates spine formation in mice and rats [18]. BAI1 acts by recruiting the cell polarity protein, Par3, and the RhoGEF, Tiam1, to synaptic sites with its C-terminal PDZ binding motif. Loss of BAI1 results in inactivated Rac1 and a reduced F-actin content in spines. The BAI3 receptor that is enriched in spines [92] is also shown to modulate spine density when activated by its ligands, C1Q-like (C1ql) proteins. Interestingly, while the knockdown of BAI3 or C1ql1 produced a significant decrease in spine density and mean spine head diameter in Purkinje cells [92], C1ql3 ostensibly promoted excitatory synapse elimination in hippocampal cultures via BAI3 [93]. This suggests that the BAI3 receptor may possess diverse signalling properties in a ligand-dependent and spatially differentiated manner. Considering the intimate relationship between cell adhesion molecules and dendritic morphology [1], it is possible that adhesion GPCRs may have a bigger role to play in structural plasticity, as more of them are deorphanised.

### 2.5. Crosstalk Between Signalling Pathways

Considering its ability to regulate brain-derived neurotrophic factor (BDNF) expression via the activation of transcription factors [94] and control neuronal differentiation [95], cAMP is indispensable in memory formation. Even though G_s_ signalling remains the key pathway that induces cAMP production, other G protein families have also been shown to partake in this process. AC2, a Ca^2+^-insensitive isoform expressed in the hippocampus [96], appears to be a nexus for the different G protein pathways, and efficiently integrates their respective signals. G_i_-coupled receptors can stimulate AC2 in the presence of an activated Gα_s_ subunit [97] or alongside a G_q_-coupled receptor [98] in a pertussis toxin-sensitive manner. Both of these observations are dependent on the release of Gβγ upon G-protein activation.

It is worth noting that the Rho GTPases can be regulated by Ca^2+^, cAMP aside. In line with the hypothesis that different Ca^2+^ sources result in opposite phenotypes of dendritic morphology, an influx of Ca^2+^ into dendrites potently activates RhoA to induce neurite retraction, whereas the release of Ca^2+^ from intracellular stores downregulates RhoA and upregulates Cdc42 that promotes actin polymerisation [99]. It is therefore tempting to wonder how this effect manifests itself with different GPCRs as those that couple to G_12/13_ typically interact with other Gα subunits as well. G_s_-coupled receptors could promote the Ca^2+^ permeability of NMDARs by PKA-mediated phosphorylation of the receptor [32]. The resultant strong and persistent elevation of Ca^2+^ could potentiate the activation of RhoA and produce the neurite retraction that we see with 5-HT_4_R activation. Receptors that are able to reduce this postsynaptic Ca^2+^ current, such as 5-HT_7_R [17], suppress RhoA and promote spinogenesis instead via Cdc42. While this has yet to be demonstrated, the elongation of spines promoted by G_q_-coupled receptors may be partially attributable to RhoA downregulation resultant from the weak, transient Ca^2+^ elevations brought about by access to the intracellular Ca^2+^ stores.

It is also possible that the induced release of intracellular Ca^2+^ stores works synergistically with the G_s_ signal. By activating the calcium-sensitive ACs, G_q_ receptors can accentuate the effect of cAMP in modulating spine morphology. AC1 and AC8 are believed to be the only Ca^2+^/calmodulin-stimulated ACs expressed in the brain, and can be found in hippocampal neurons [100]. Mutant mice with double knockout of AC1 and AC8 do not exhibit late-LTP and long-term memory formation, but memory formation could be rescued by hippocampal delivery of forskolin [100]. Conversely, cAMP is also able to modulate the release of Ca^2+^ from intracellular stores through the inositol 1,4,5-trisphosphate receptor (IP_3_R). IP_3_R1, in particular, is enriched in the hippocampus [101], and its role in synaptic plasticity is highlighted by data suggesting that LTP is facilitated in IP_3_R1 knockout mice [102]. This IP_3_R subtype is phosphorylatable by PKA, and the phosphomodification enhances receptor sensitivity towards IP_3_ to potentiate Ca^2+^ transients evoked by the ligand [103].

The studies on 5-HT_4_R [16], 5-HT_7_R [17] and S1PR2 [28,104] have also revealed intriguing coincidences about their signalling properties. All three GPCRs can couple to the G_s_ and G_12/13_ subunits and play a part in spine morphogenesis. It has indeed been shown previously that cAMP does regulate the activation of small GTPases [105,106], thereby providing legitimacy to the possibility of crosstalk between G_s_ and G_12/13_ signalling. This may warrant further investigation into the integrated effects of the G_s_/G_12_ and G_s_/G_13_ combinations, while taking into account changes in the Ca^2+^ permeability of ion channels resultant from receptor activation.

Insight into receptor dimerisation may also open up new avenues for exploring the role of GPCRs in spine morphogenesis, as ligand-binding and signalling properties could be altered upon the formation of heteromers. It is noteworthy that the G_s_-coupled A_2A_R has been suggested to dimerise with D_2_R [107] and CB_1_R [108], both G_i_-coupled receptors that negatively impact on spine growth. Through these receptor dimers, adenosine is able to antagonistically modulate signalling initiated by dopamine [109] and cannabinoids [110], thereby rendering A_2A_R a potential candidate for manipulating the structural plasticity processes.

## 3. Concluding Remarks

As GPCRs lie upstream of processes that produce key intracellular second messengers such as cAMP and Ca^2+^, it does not come as a surprise that such receptors should form part of the regulatory mechanisms for dendritic spine morphology. While scant evidence exists for the G_i_ family, substantial support is available for a role of G_q_- and G_12/13_-coupled receptors in spine morphogenesis. Curiously, studies on G_s_-coupled receptors are relatively scarce, despite the fact that the activation of PKA and CREB are linked to structural plasticity. As many GPCRs can activate more than a single G protein class, potential crosstalk between G_q_/G_12/13_-, G_s_/G_12/13_- and G_s_/G_q_-coupled receptors may significantly affect their ability to modulate structural plasticity. The extensive overlap of pathways governing actin dynamics, synaptic transmission and G protein signalling suggests ample potential for a role of GPCRs in structural plasticity. Based on their indicative role in regulating synaptic plasticity and abundant expression in the hippocampus, GPCRs belonging to the adenosine [111], histamine [112], cholinergic [113], somatostatin [114] and GABA_B_ [115] receptor families may be involved in structural plasticity. This list may grow further as the functions of the 15 orphan GPCRs that are highly expressed in the hippocampus are revealed [14]. Even though this review focuses primarily on brain areas tightly linked to neural plasticity, it is important to stress that GPCRs do extend their influence on spine morphology beyond the hippocampal and cortical regions, not least into the cerebellum [92], nucleus accumbens [116] and striatum [117]. Further, recent research findings invite efforts that venture beyond conventional G protein signalling, as more and more unorthodox mechanisms through which the receptors direct actin dynamics are being uncovered.

In addition to memory formation, the basis of many neurological diseases, including Fragile X syndrome and autism-related disorders, can be traced back to aberrant dendritic spine morphology. The identification of GPCRs that act upstream of spine morphogenesis may assist the discovery of druggable targets and the development of medical interventions for such conditions.

## Figures and Tables

**Table 1:** Selected G protein-coupled receptors (GPCRs) regulating structural plasticity in the hippocampus and cerebral cortex.

Receptor	Mechanism		Receptor Activation			Receptor Knockdown		Ref.
		Spine Density	Spine Morphology	Ion Channel Expression	Spine Density	Spine Morphology	Ion Channel Expression	
**5-HT_2A_R**	Kalirin-7/Rac	--	Spine area, length & breadth increases	--	--	--	--	[15]
**5-HT_4_R**	G_s_	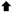	--	--	--	--	--	[16]
**5-HT_7_R**	G_12_	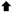	--	AMPAR increases	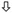	--	--	[17]
**BAI1**	Par3/Tiam1/Rac1	--	--	--	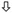	Spine length increases Spine diameter decreases Filopodial spine density increases	--	[18]
**CB_1_R**	G_i_; WAVE1/Rac1	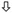	Mushroom spine density decreases	--	--	--	--	[19]
**D_2_R**	G_i_	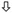 ^a^	Mushroom & thin spine lengths increase while densities decrease Filopodium density increases ^a^	NMDAR (GluN2B) decreases	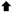 ^c^	--	--	[20]
**Fzd9**	Gβγ; G_o_	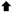	Spine head width increases	--	--	--	--	[21]
**MC4R**	G_s_	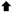	Mature spine density increases	AMPAR (GluA1) increases	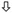	Spine volume decreases Mature spine density decreases Immature spine density increases	--	[22]
**mGluR1/5**	G_q_	--	Spine length increases Filopodial & non-mushroom spine density increases	--	--	--	--	[23]
**MOR**	G_i_	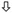 ^b^	--	--	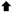 ^d^	--	--	[24,25]
**PAR1**	G_q_; β-arrestin	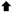	--	--	--	--	--	[26]
**S1PR2**	G_13_	--	Spine length decreases Spine head width decreases	--	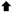 ^e^	--	AMPAR increases ^f^	[27,28]

^a^ Overexpression and receptor activation; ^b^ chronic receptor activation; ^c^ similar effect observed with prolonged receptor blockade; ^d^ similar effect observed with receptor blockade; ^e^ receptor blockade; ^f^ agonist scavenging. 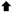 = increase; 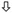 = decrease.
